# CPT11 with P-glycoprotein/CYP 3A4 dual-function inhibitor by self-nanoemulsifying nanoemulsion combined with gastroretentive technology to enhance the oral bioavailability and therapeutic efficacy against pancreatic adenocarcinomas

**DOI:** 10.1080/10717544.2021.1989087

**Published:** 2021-10-18

**Authors:** Ling-Chun Chen, Wei-Jie Cheng, Shyr-Yi Lin, Ming-Tse Hung, Ming-Thau Sheu, Hong-Liang Lin, Chien-Ming Hsieh

**Affiliations:** a Department of Biotechnology and Pharmaceutical Technology, Yuanpei University of Medical Technology, Hsinchu, Taiwan; b School of Pharmacy, College of Pharmacy, Taipei Medical University, Taipei, Taiwan; c Division of Gastroenterology, Department of Internal Medicine, Taipei Medical University, Wan Fang Hospital, Taipei, Taiwan; d Department of General Medicine, School of Medicine, College of Medicine, Taipei Medical University, Taipei, Taiwan; e School of Pharmacy, College of Pharmacy, Kaohsiung Medical University, Kaohsiung, Taiwan

**Keywords:** CPT11, SN-38, silymarin, self-nanoemulsifying nanoemulsion, GRDDS, MIA CaPa-2

## Abstract

Therapeutic efficacies of orally administrated hydrophobic chemodrugs are decreased by poor water solubilities and reduced oral bioavailabilities by P-glycoprotein (P-gp) and CYP450. In this study, CPT11 alone or combined with dual-function inhibitors (baicalein (BA) silymarin (SM), glycyrrhizic acid (GA), and glycyrrhetinic acid (GLA)) of P-gp and CYP450 loaded in a lecithin-based self-nanoemulsifying nanoemulsion preconcentrate (LBSNENP) to improve the solubility and inhibit the elimination by P-gp and CYP450. Results revealed that the LBSNENP composed of Capryol 90, lecithin/Tween 80/Cremophor EL, and propylene glycol at a weight ratio of 18:58:24 (designated PC90C10P0) was optimally selected. Encapsulating CPT11 with PEO-7000K in PC90C10P10/30 further enhanced the resultant hydrogel to be gastro-retainable and to release CPT11 in a sustained manner. Pharmacokinetic study of CPT11-loaded PC90C10P0 administered orally revealed an absolute bioavailability (FAB, vs. intravenous CPT11) of 7.8 ± 1.01% and a relative bioavailability (FRB1, vs. oral solution of CPT11) of 70.7 ± 8.6% with a longer half-life (*T*_1/2_) and mean residence time (MRT). Among the dual-function inhibitors, SM was shown to be the most influential in increasing the oral bioavailability of CPT11. SM also increased the plasma concentration of the SN-38 active metabolite, which formed from the enhanced plasma concentration of CPT11. It is concluded that treatment with CPT11 loaded in PC90C10P0 with or without solubilization with SM could expose tumors to higher plasma concentrations of both CPT11 and SN-38 leading to enhancement of tumor growth inhibition with no signs of adverse effects.

## Introduction

Cancer is one of the most devastating diseases and a leading cause of death in the world. According to the mortality data from the National Center for Health Statistics in 2013, one in four deaths in the United States was due to cancer (Siegel et al., 2013). Various anticancer drugs have been successfully developed in the last decade. Among those drugs, oral chemotherapy for the treatment of cancer offers many patients more-convenient and less-invasive treatment options compared to intravenous (i.v.) administration. Oral chemotherapy can also enable the development of dosing regimens and results in prolonged periods of plasma concentration above pharmacologically relevant levels (Veltkamp et al., 2006; Goodin, 2007).

However, oral chemotherapy is still a great challenge as major anticancer drugs are poorly soluble in water, resulting in a low effective concentration and limited absorption in the gastrointestinal (GI) tract. The expression of ABC efflux transporters, like P-glycoprotein (P-gp) and drug metabolizing enzymes, like cytochrome P450 3A (CYP 3A), in the lumen of the GI tract often limits their oral absorption (Sparreboom et al., 1997; Yang et al., 2004). In addition, oral administration is also subject to a ‘first-pass effect’ when the absorbed drug is first transported to the liver for extraction and metabolism via the hepatic portal vein. Therefore, many antineoplastic agents used in chemotherapy are administered by i.v. to patients to bypass problems of absorption and presystemic metabolism.

Irinotecan (CPT11) is a camptothecin derivative that has demonstrated anticancer activities in many solid tumors. Presently, it is widely used to treat colorectal, pancreatic, and lung cancer. CPT11 is currently mainly administered by an i.v. bolus injection. Nevertheless, it was shown in an animal model that a lower dose by daily administration of CPT11 is as effective as and less toxic than less-frequent higher dose administration (Houghton et al., 1995; Thompson et al., 1997). The greater efficacy of extended-duration therapy and the reduced toxicity of lower dose daily administration make CPT11 an excellent candidate for oral delivery as a convenient way of achieving protracted lower dose schedules (Rothenberg, 1998).

The oral bioavailability of CPT11 is reported to be low (Kuhn, 1998; Drengler et al., 1999) and highly variable (Schoemaker et al., 2005; Soepenberg et al., 2005). Following oral administration, metabolizing enzymes of CYP3A convert CPT11 to the inactive metabolites of APC and NPC, while drug transporters of P-gp (ABCB1) can pump out of absorbed CPT11 into the lumen of the GI tract, both of which result in significant reductions in the oral bioavailability. Upon being taken up by enterocytes, CPT11 is metabolized into its major active (100–1000 times more active) metabolite, SN-38, with the help of carboxylesterases that are located in enterocytes, but with only a fraction of the CPT11 being directly converted into SN-38, because a competing process exists in the CYP3A oxidation of CPT11 into the inactive metabolites of APC and NPC. Once entering the liver, CPT11 is still metabolized into SN-38 by carboxylesterases located in hepatocytes, but it is competitively oxidized by CYP3A into the inactive metabolites of APC and NPC. On the other hand, SN-38 is inactivated in the liver via glucuronidation to SN-38G by several uridine diphosphate glucuronosyltransferase subfamily 1A (UGT1A) isoforms, with UGT1A1 being the most important (Rivory & Robert, 1995; Haaz et al., 1997). Several drug transporters are involved in eliminating CPT11, SN-38, and SN-38G that accumulate in the liver. The clearance of CPT11 is mainly biliary (66%) and is transported into the bile by P-gp (ABCB1) and the ATP-binding cassette drug-transporter C2 (ABCC2) (Slatter et al., 2000; Mathijssen et al., 2001). SN-38 is transported into the bile by ABCB1, ABCC2, and ATP-binding cassette drug-transporter G2 (ABCG2, also known as breast cancer resistance protein (BCRP)), while SN-38G can be transported into the bile by ABCC2 and ABCG2. From the bile, all three are then secreted into the intestines along with bile juice. In the intestines, SN-38G can be de-glucuronidated into SN-38 by β-glucuronidase-producing bacteria, which can result in enterohepatic circulation of SN-38 (Cole et al., 1985; Fujisawa & Mori, 1997; Sperker et al., 1997; Younis et al., 2009), and SN-38 so-obtained is also principally responsible for the gastrointestinal toxicity of CPT11 (Takasuna et al., 1996; Kong et al., 2014).

Taken together, P-gp inhibition in both the intestines (ABCB1) and bile (ABCB1, ABCC2, ABCG2, and BRCP) eliminates the first-pass effect, resulting in increased oral absorption and systemic exposure to CPT11 and SN-38. Diarrhea can also be ameliorated due to inhibition of biliary excretion of both the SN-38 and SN-38G metabolites causing decreased accumulation. CYP3A inhibition by both enterocytes and hepatocytes decreases the competing metabolism of CPT11 into the inactive APC and NPC metabolites, while potentially increasing the formation of SN-38 by carboxylesterases, resulting in increased systemic exposure to SN-38 which enhances the tumor inhibition efficacy. Recently, a complex drug–drug interaction (DDI) of CPT11 with the involvement of many metabolizing enzymes and P-gp transporters was reviewed and revealed that an important DDI between CPT11 and the combination treatment with ritonavir and lopinavir caused by CYP3A4, UGT1A1, and ABC transporter inhibition resulted in greater than a twofold increase in SN-38 area under the concentration-time curve (AUC) and a 36% decrease in the SN-38G/SN-38 AUC ratio (Femke et al., 2018). Overall, it is expected that the oral delivery of CPT11 in combination with the dual P-gp/CYP3A function inhibitor would be beneficial to the antitumor efficiency as a result of enhancing the oral bioavailability of CPT11 and the formation and accumulation of the SN-38 active metabolite.

Furthermore, both CPT11 and SN-38 can exist in a closed ring lactone form and an open, hydroxy acid form. Only the lactone form of either compound is active against tumors (Stewart et al., 1997; Drengler et al., 1999). If CPT11 can be released in the stomach, the low gastric pH will keep more of the CPT11 in the active lactone form. Therefore, more of the SN-38 that is produced by carboxylesterases in the gut should be in the active lactone form (Stewart et al., 1997; Drengler et al., 1999). This assumption of a higher ratio of active SN-38 to inactive SN-38 by oral delivery was borne out in an animal model and a phase I study (Kuhn, 1998; Zamboni et al., 1998; Drengler et al., 1999). Delivery and absorption of CPT11 preferentially in the stomach should improve its oral systemic bioavailability against tumor cells by increasing the proportion of SN-38 that reaches the tumor in active form. Therefore, the oral delivery of CPT11 using a gastroretentive drug delivery system (DDS; GRDDS) to locally release CPT11 in an acidic condition of stomach would be beneficial for the therapeutic efficacy. Furthermore, the oral delivery of CPT11 using a GRDDS would also prevent CPT11 from transiting to the lower GI tract, whereby avoiding efflux by P-gp to reduce its bioavailability.

Recently, increasing accumulating evidence has demonstrated that non-cytotoxic naturally occurring dietary and herbal components are capable of interacting with both CYP3A metabolizing enzymes and P-gp transporters (Cho et al., 2011; Yang et al., 2015). Among them, silymarin, a flavonoid complex extracted from seeds of the milk thistle, is able to inhibit CYP3A4, UGT1A1, and ABC transporters (van Erp et al., 2005; Mirkov et al., 2007; Lin et al., 2008). Baicalein, the major flavonoid in Scutellariae radix, was reported to modulate the CYP3A subfamily and/or P-gp (Cho et al., 2011; Li et al., 2011). An *in vitro* study reported that glycyrrhizic acid (GA) inhibited the function of P-gp, in a similar way to glycyrrhetinic acid (GLA), a major metabolite of GA (Yoshida et al., 2006). Moreover, it was also reported that GLA is an inhibitor of CYP3A, CYP1A1, and CYP2E1 in rat liver microsomes (Yang et al., 2001; Nabekura et al., 2008; Tu et al., 2010). Therefore, all four potential dual-function inhibitors for CYP 3A and P-gp were selected to examine their effects on the oral bioavailability of CPT11 in this study.

Nevertheless, the poor water solubilities of CPT11 and the four dual-function inhibitors are still a great challenge for oral delivery achieving a desired effective concentration for therapy. SMEDDSs are one of the most successful nano-range DDSs, which include pre-concentrates of oils, a surfactant mixture, a cosurfactant, and a drug. On dilution with GI fluid, the preconcentrates self-microemulsify into nano-range oil droplets containing drug molecules (Pouton, 2000). SMEDDSs require high surfactant/cosurfactant concentrations to reduce the surface tension between the oil and water phases and achieve zero interfacial tension, thus leading to increased toxicity (Lawrence & Rees, 2000). From this perspective, lecithin-based SMEDDSs are especially desirable since lecithin is a naturally occurring nontoxic biological surfactant (Yuan et al., 2008), as a kind of phospholipid that functions as a crucial component of the cell membrane to maintain membrane fluidity and an absorption enhancer to facilitate drug absorption (Jin et al., 2013). Negi et al. (2013) reported that a SMEDDS formulation of CPT11 with excipients having P-gp modulation activity resulted in significantly increased oral bioavailability (approximately 4-fold), indicating that it is a promising way to orally deliver CPT11 and a dual-function inhibitor by lecithin-based SMEDDSs by enhancing the oral bioavailability of CPT11 and the formation and accumulation of the SN-38 active metabolite.

The development of lecithin-based self-nanoemulsifying nanoemulsion preconcentrates (_LB_SNENPs) to load CPT-11 and four dual-function inhibitors for oral delivery of resultant self-nanoemulsifying nanoemulsions (_LB_SNENAs) with the potential to enhance the oral bioavailability was adopted from those previous reports and optimized in this study (Lin et al., 2011, 2013; Chen et al., 2016; Su et al., 2018). Furthermore, most SMEDDSs, as exemplified as _LB_SNENA in this study, are thermodynamically stable liquid formulations with a high solubilization capacity for poorly water-soluble drugs, and because of that, they need to be filled directly into soft- or hard-gelatin capsules for convenient oral administration. Considering that it is necessary to encapsulate the liquid of CPT11/dual-function inhibitor-containing _LB_SNENA preconcentrates (_LB_SNENPs) into soft- or hard-gelatin capsules, a GRDDS in capsule dosage form, which is contrary to traditional tablet dosage forms, was also developed and optimized in this study for the efficient oral delivery of CPT11.

## Materials and methods

### Materials

Baicalein (BA; at 95%), silymarin (SM; at 80%), glycyrrhizic acid (GA; at 95%), and glycyrrhetinic acid (GLA; at 95%) were purchased from Sanjaing (Jiaxing, China). Irinotecan hydrochloride (CPT11) was provided by Qilu Pharm (Jinan, China). SN-38 was delivered by Scino Pharm (Tainan, Taiwan). Capryol-90 was procured from Gattefosse (Lyon, France), and Tween 80 was supplied by Merck (Darmstadt, Germany). Soybean lecithin (Lipoid S-100) was purchased from Lipoid (Ludwigshafen, Germany). All reagents for high-performance liquid chromatography (HPLC) or ultra-performance liquid chromatographic (UPLC)/tandem mass spectrometric (MS/MS) analyses were of an HPLC or MS grade, and other reagents were of an analytical grade.

### Methods

#### Construction of _LB_SNENP phase diagrams and optimization

Based on a preliminary study of the solubility and emulsification tests, Capryol-90 was selected as the oil phase, a mixture composed of lecithin and Tween 80 with or without Cremophor-EL was selected as the surfactant system (SAA), and propylene glycol (PG) was chosen as the cosurfactant. The boundaries of the nanoemulsion domains were determined using a pseudo-ternary phase diagram. Each component indicated the apex of a triangle. A series of blank _LB_SNENP formulations was prepared for each of the three components using varying concentrations of Capryol-90, SAA, and PG. For any mixture, the total weight% of the three components always added up to 100%. The efficiency of nanoemulsion formation was assessed by adding 100 μL of each mixture to 10 mL of distilled water and gently stirring with a magnetic stirrer. A visual observation was made to identify the spontaneity of self-nanoemulsification. The formulations whose dilution showed phase separation or coalescence of oil droplets were judged to be poor self-microemulsifying formulations, while those that were capable of forming a clear, uniform nanoemulsion were chosen to construct the self-nanoemulsifying region. Droplet sizes of those nanoemulsions were also determined using photon correlation spectrometry to objectively confirm the apparent spontaneity of the nanoemulsion. The self-nanoemulsifying region was adopted for optimization to choose potential _LB_SNENP formulations for encapsulating CPT11 and the four dual-function inhibitors.

#### Evaluation of swellable/floating GRDDSs in capsule form

In a previous study (Lin et al., 2020), it was found that swellable/floating GRDDSs in capsule form could be simply prepared by filling various amounts of PEO-7000K into 00-sized capsules. Soon after contacting simulated gastric fluid, the swelling capacity of the PEO-7000K hydrogel increased with an increasing amount of PEO-7000K initially, then decreased with a further increase in the PEO-7000K amount. Apparently, with 20–40% of PEO-7000K, the hydrogel could swell to a size that was large enough to prevent it from passing through the pylorus and also caused it to float in the medium. Therefore, novel oral delivery systems combining swellable/floating GRDDSs with _LB_SNENPs in a 00-sized capsule were simply produced by filling capsules with 10%, 30%, and 50% wt/wt of _LB_SNENP and PEO-7000K (designated PC_90_C_10_P_10_, PC_90_C_10_P_30_, and PC_90_C_10_P_50_, respectively).

#### *Physicochemical characterization of*_LB_*SNENAs*

After self-nanoemulsifying _LB_SNENPs were placed in double-distilled (DD) water, the average particle size and size distribution of the so-obtained _LB_SNENAs were measured at a scattering angle of 90° with an N5 submicron particle size analyzer (Beckman Coulter, Brea, CA) at 25 °C, and the intensity autocorrelation of the sample was in a range of 5 × 10^4^–10^6^. The average diameter and polydispersity index (PDI) of three measurements were reported. The solubilities of CPT11, BA, SM, GA, and GLA in the optimal _LB_SNENP were detected with a validated ultraviolet (UV) or HPLC method. HPLC conditions for CPT11 were as follows: Biosil Aqu-ODS-5 µm (C18, 4.6 × 250 mm, Biotic Chemical, Taipei, Taiwan); composition of the mobile phase was phosphate buffer (pH 3 ± 0.05)/acetonitrile/tetrahydrofuran (THF) (60/30/2 vol/vol); the flow rate was 0.8 mL/min; and the fluorescence was detected with an excitation wavelength of 370 nm and emission wavelength of 470 nm. UV spectrophotometric absorbance measurements were used to detect BA, SM, GA, and GLA at respective UV wavelengths of 287, 287, 248, and 248 nm, in samples after 5-fold dilution with methanol. Each data point is the mean of at least three individual trials. The assay method was validated before implementation.

#### *In vitro release of CPT11 and four dual-function inhibitors from optimal*_LB_*SNENPs*

The USP dissolution apparatus 2 (model VK7020, Vankel, Cary, NC) was used to measure the release of CPT11 and four dual-function inhibitors from optimal _LB_SNENPs at an agitation speed of 100 rpm in simulated gastric fluid (SGF) without enzymes (pH 1.2). The temperature of the dissolution medium was maintained at 37 ± 0.5 °C. Aliquots of 5 mL of sample were withdrawn for the assay at predetermined time intervals and replaced with the same volume of fresh medium. Contents of CPT11, BA, SM, GA, and GLA were determined as aforementioned. Each dissolution data point is the mean of at least three individual trials.

#### In vivo pharmacokinetic (PK) studies in rabbits

All animal experiments were carried out in accordance with a protocol approved by the Laboratory Animal Center of Taipei Medical University (approval no: LAC-2015-0108) and conducted in compliance with the Taiwanese *Animal Welfare Act*. First of all, New Zealand white rabbits weighing ∼3 kg were utilized to investigate the PK profiles of CPT11 and its active metabolite, SN-38, after oral administration of CPT11 (40 mg/rabbit) solubilized in DD water (solution), in _LB_SNENPs (PC_90_C_10_P_0_), and in _LB_SNENPs containing 10% and 30% PEO-7000K (PC_90_C_10_P_10_ and PC_90_C_10_P_30_), or CPT11 (40 mg/ rabbit) in _LB_SNENPs (PC_90_C_10_P_0_) combined with each of four dual-function inhibitors (80 mg/ rabbit) (PC_90_C_10_P_0_/BA, PC_90_C_10_P_0_/SM, PC_90_C_10_P_0_/GA, and PC_90_C_10_P_0_/GLA), or CPT11 (40 mg/ rabbit) combined with SM (80 mg/ rabbit) in _LB_SNENPs containing 10% PEO-7000K (PC_90_C_10_P_10_). All blood samples from the right ear vein were collected in heparinized tubes before dosing and at 0.0833, 0.5, 1, 2, 3, 4, 6, 8, 10, 12, 24, 36, 48, and 72 h after administration. I.V. administration of CPT11 (4 mg/ rabbit) in water for an injection was used as the control for calculating the absolute oral bioavailability (*F*_AB_). All blood samples were immediately centrifuged at 3000 rpm for 15 min at 4 °C to obtain plasma. Plasma samples were stored at −80 °C before the high-performance liquid chromatographic (HPLC) analysis as described below. PK parameters are presented as the mean and standard deviation (SD) from individual rabbits in each group and were estimated through a noncompartmental analysis. The terminal elimination rate constant (*K*_e_) was estimated from the slope of the log-linear phase of declining plasma concentrations of an alendronate versus time graph. The half-life (*T*_1/2_) was calculated using the following equation: *T*_1/2_ = ln_2_/*K*_e_. The area under the concentration-time curve from beginning to the last time point (AUC_0→last_) was calculated using the trapezoidal method. Summation of AUC_0→last_ and the concentration at the last measured point divided by Ke yielded AUC_0→∞_. Clearance (CL) was calculated by dividing the dose by AUC_0→∞_, and the volume of distribution (V) was determined by dividing CL by *K*_e_. The absolute bioavailability (*F*_AB_) and relative bioavailability (*F*_RB_) were respectively calculated according to Equations (1) and (2), respectively

(1) $$F_{AB}=\frac{{\left[\mathrm{AUC}\right]}_{po}D_{IV}}{{\left[\mathrm{AUC}\right]}_{IV}D_{po}}\times 100$$

where [AUC]*_po_* and [AUC]*_IV_* are the area under the plasma concentration curves (AUCs) after oral and intravenous administration. *D_IV_* and *D_po_* are the doses after oral and intravenous administration, respectively.

(2) $$F_{RB}=\frac{{\left[\mathrm{AUC}\right]}_{A}D_{B}}{{\left[\mathrm{AUC}\right]}_{B}D_{A}}\times 100$$

where AUC for a _LB_SNENPs formulation [*AUC*]*_A_* and the AUC for the same drug in solution [*AUC*]*_B_* after oral administration were compared. *D_B_* and *D_A_* are the doses for solution and _LB_SNENPs formulation, respectively.

#### Plasma analysis of CPT11 and SN-38 by an HPLC method

The procedure for CPT11 and SN-38 extraction from plasma was as follows. Plasma (200 μL) was vigorously mixed with 1.4 mL ethyl acetate for 10 min to extract CPT11 and SN-38. After centrifugation at 13,000 rpm for 30 min at 4 °C, 1.2 mL of ethyl acetate was collected, and then subjected to evaporation under N_2_ gas at 50 °C. The mobile phase (200 μL) was added to reconstitute the dried residual, vortexed for 5 min, and then centrifuged at 10^4^ rpm and 25 °C for 3 min. The supernatant (180 µL) was collected, and 50 µL was injected into the HPLC system for analysis. HPLC conditions for CPT11 and SN-38 were as follows: the column was Biosil Aqu-ODS-5 µm (C18, 4.6 × 250 mm, Biotic Chemical, Taipei, Taiwan); composition of the mobile phase was phosphate buffer (pH 3 ± 0.05)/acetonitrile/THF (65/35/2 vol/vol); the flow rate was 0.8 mL/min; the column oven temperature was set to 40 °C; and fluorescence detection used an excitation wavelength of 370 nm for both CPT11 and SN-38 and emission wavelengths of 470 nm for CPT11 and 534 nm for SN-38.

#### Tumor inhibition studies

All animal experiments were carried out in accordance with a protocol approved by the Laboratory Animal Center of Taipei Medical University (approval no: LAC-2016-0287), and all experiments were performed in accordance with animal care guidelines. All Balb/c mice received a subcutaneous injection of 100 µL (containing 5 × 10^6^ cells) of the MIA PaCa-2 cell suspension in Matrigel into the right thigh. These tumor-bearing mice with around 100-mm^3^ tumor volumes were randomized into five groups: a control group (phosphate-buffered saline (PBS)) and four groups including i.v. administration of a CPT11 solution, oral administration of CPT11 alone in water by an injection in _LB_SNENPs (PC_90_C_10_P_0_), and CPT11 combined with SM in _LB_SNENPs (PC_90_C_10_P_0_) containing 10% PEO-7000K (PC_90_C_10_P_10_). Each formulation was orally administered once every 3 days for 12 days. The tumor volume was calculated by the modified ellipsoidal formula of 1/2 length×width^2^. Mice body weights (BWs) and tumor volumes were measured every 3 days after the injection. Mice were sacrificed by CO_2_, and the tumors were harvested and weighed on day 21. The tumor growth inhibition rate (TGI%) was calculated according to Equation (3)

(3) $$\left(W_{c}-W_{t}\right)/W_{c}$$

where *W*_c_ is the tumor weight of the control group and *W*_t_ is the tumor weight of each formulation group.

### Statistical analysis

Data are presented as the mean ± standard deviation (SD) of each group. The significance among samples was assessed by a one-way analysis of variance (ANOVA). Significant differences between groups were indicated by *p < .05, **p < .01, and ***p < .001.

## Results and discussion

### Construction and optimization of _LB_SNENPs

**Figure 1.**
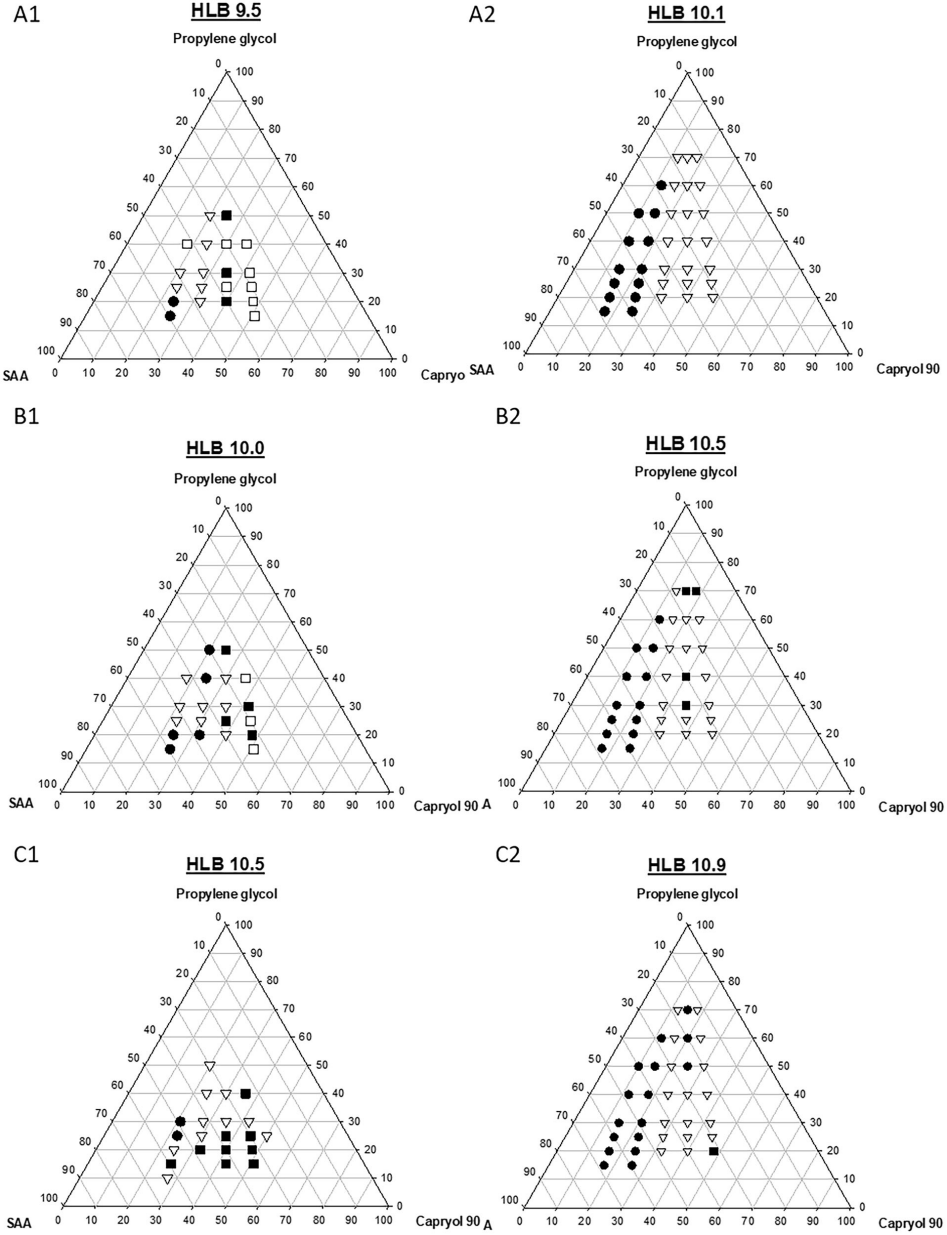
A pseudo-ternary phase diagram for _LB_SNENP and the influence of the hydrophilic-lipophilic balance (HLB) value of SAA on the formation of self-nanoemulsifying nanoemulsion was compared. (A1–C1) composed of lecithin/Tween 80 at 2.75%/2.75% wt/wt, 2.5%/3.0% wt/wt, and 2.25%/3.25% wt/wt, respectively, and with HLB values of 9.5, 10.0, and 10.5, respectively. (A2–C2) were composed of lecithin/Tween 80/Cremophor EL at 2.75%/2.75%/1.1% wt/wt, 2.5%/3.0%/1.1% wt/wt, and 2.25%/3.25%/1.1% wt/wt, and with HLB values of 10.1, 10.5, and 10.9, respectively. The labels for solid circle (•), upside down triangle (▼), solid square (■), and open square (□) were designated as the particle size after self-nanoemulsifying measured to be <200, 200–250, 20–300, and 300–350 nm, respectively. Each point represents the mean ± S.D. of three determinations (*n* = 3).

A pseudo-ternary phase diagram for _LB_SNENPs was constructed using Capryol-90 as the oil phase, lecithin/Tween 80/Cremophor EL as the surfactant (SAA), and propylene glycol (PG) as the cosurfactant in a drug-free condition, and results of the appearance and particle size are illustrated in Figure 1. The influence of the HLB value of the SAA on the formation of self-nanoemulsifying nanoemulsions was compared, in which Figure 1(A1–C1) is composed of lecithin/Tween 80 at 2.75%/2.75% wt/wt, 2.5%/3.0% wt/wt, and 2.25%/3.25% wt/wt, respectively, and with hydrophilic-lipophilic balance (HLB) values of 9.5, 10.0, and 10.5, respectively, while Figure 1(A2–C2) is composed of lecithin/Tween 80/Cremophor EL at 2.75%/2.75%/1.1% wt/wt, 2.5%/3.0%/1.1% wt/wt, and 2.25%/3.25%/1.1% wt/wt, and with HLB values of 10.1, 10.5, and 10.9, respectively. Based on observations during the preparation, it was found that when the weight % of Capryol 90 was <15% of the total amount of the _LB_SNENP, a longer time was required (∼8 h) to completely dissolve to form a clear yellowish liquid, but it was even necessary to immerse the formulation in a water both at a temperature of 55–60 °C. Furthermore, the resulting _LB_SNENPs became a viscous gel after being cooled to room temperature, and the so-obtained viscous gel was not easier to disperse in water for self-nanoemulsification. Even after being subjected to a high intensity of vortexing to aid dispersion, it was only able to form a milky-white emulsion. On the contrary, when the weight % of Capryol 90 was >15%, the necessary time to completely dissolve decreased with an increasing weight % of Capryol 90 at a heating temperature of 50–55 °C and the time to dissolve was further shortened by increasing the weight % of PG. Furthermore, most of the so-obtained _LB_SNENP remained a clear transparent light-yellowish liquid after being cooled to room temperature and was able to solubilize in the water for self-nanoemulsifying to form self-nanoemulsifying nanoemulsions with a high degree of transmittance.

**Figure 2.**
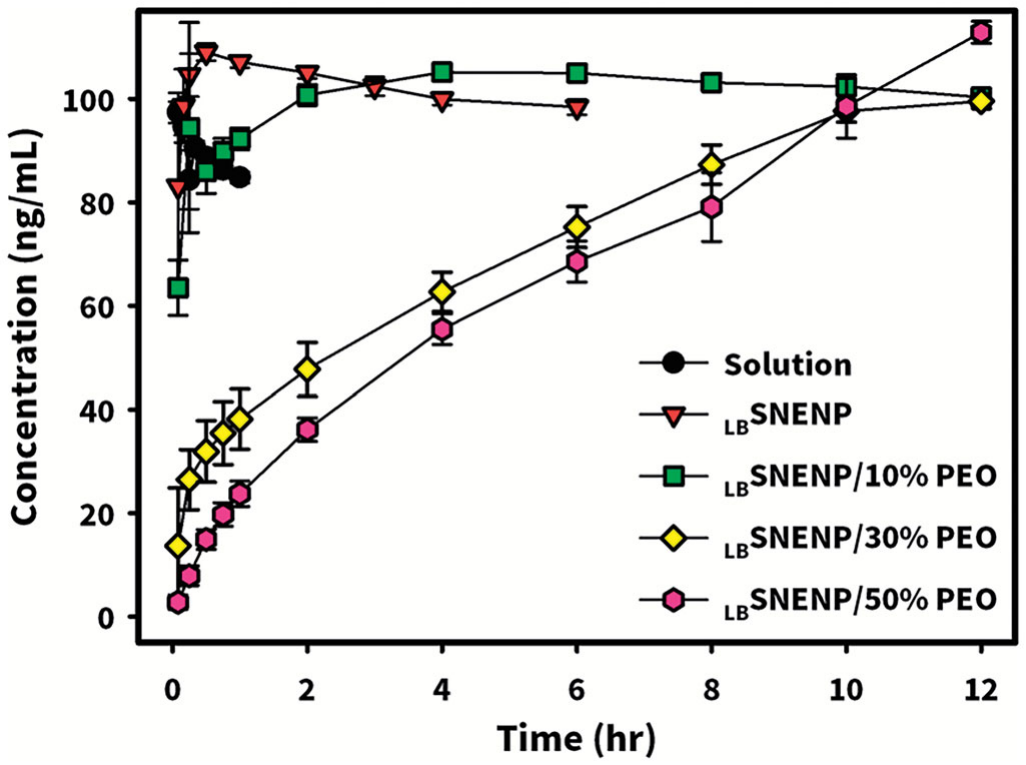
*In vitro* dissolution profiles of CPT11 (40 mg/g) from PC_90_C_10_P_0_, PC_90_C_10_P_10_, PC_90_C_10_P_30_, and PC_90_C_10_P_50_, which were composed of 0%, 10%, 30%, and 50% wt/wt, respectively, of PEO-7000K (with respect to the weight of PC_90_C_10_P_0_) and filled into 00-sized capsules. Each point represents the mean ± S.D. of three determinations (*n* = 3).

Furthermore, as Figure 1(A1–C1) reveals, there was a trend of a decreasing droplet size of the nanoemulsion with an increase in the weight % of Tween 80 in the SAA formulation. Nevertheless, these nanoemulsions were observed to be unstable at room temperature, showing various extents of creaming and precipitation. Profoundly, with the addition of Cremophor EL to three SAA systems as shown in Figure 1(A2–C2), regardless of which ratio was used, all had a droplet size smaller than 250 nm, and the resulting nanoemulsion had much improved stability with no creaming or precipitation.

As shown in Figure 1(C2), the addition of Cremophor EL to the SAA of _LB_SNENPs could form nanoemulsions with a droplet size of <250 nm and excellent stability. Among them, those _LB_SNENPs containing a low ratio of Capryol 90 to SAA composed of lecithin, Tween 80, and Cremophor EL at a 2.25%:3.25%:1.1% wt/wt ratio with an HLB value of 10.9 showed exceptional physical characteristics. An optimized _LB_SNENP (PC_90_C_10_P_0_) composed of Capryol 90, SAA, and PG at a weight ratio of 18:58:24 was selected as the appearance of the resultant nanoemulsion by self-nanoemulsifying PC_90_C_10_P_0_ containing 10 mg/g of CPT11 was observed to be a transparent bluish without creaming in a 30-day period at room temperature, while the mean droplet size and PDI for that were determined to not differ from those on day 0. Furthermore, the loading amount measured as the solubilities of CPT11, BA, SM, GA, and GLA in 1 g of PC_90_C_10_P_0_ were determined to be 40, 80, 130, 200, and 80 mg/g resulting in so-obtained nanoemulsions after self-nanoemulsifying with mean droplet sizes (nm) and PDI values of 157.3 ± 2.08 and 0.665 ± 0.020, 171.0 ± 6.52 and 0.863 ± 0.087, 247.7 ± 10.97 and 0.553 ± 0.073, 102.1 ± 0.67 and 0.602 ± 0.031, and 143.5 ± 0.04 and 0.559 ± 0.063, respectively, compared to values for the drug-free nanoemulsion of 158.7 ± 1.66 and 0.603 ± 0.017. This optimized PC_90_C_10_P_0_ formulation was selected for a further optimization study of GRDDSs below.

### Optimization of swellable/floating GRDDSs in capsule form

Based on a previous study (Lin et al., 2020), PEO-7000K presented in a nilotinib-loaded GRDDS formulation was found to be able to make a capsule form of GRDDS which swelled to a size larger than the diameter of the pylorus after exposure to simulated gastric acid leading to a resultant floating hydrogel in the stomach for a longer period of time to sustain the release of nilotinib. To maintain the release of CPT11 in the stomach’s acidic environment to increase the *in vivo* stability and prevent the pumping out of absorbed CPT11 into the lumen of the GI tract by P-gp (ABCB1) located on the apical side of enterocytes leading to enhancement of oral bioavailability, oral delivery systems combining swellable/floating GRDDS with optimized PC_90_C_10_P_0_ formulation in capsule form were produced by filling 10%, 30%, and 50% wt/wt of PEO-7000K (with respect to the weight of PC_90_C_10_P_0_) with PC_90_C_10_P_0_ into 00-sized capsules, which were respectively designated as PC_90_C_10_P_10_, PC_90_C_10_P_30_, and PC_90_C_10_P_50_. The dissolution profiles of CPT11 from PC_90_C_10_P_0_, PC_90_C_10_P_10_, PC_90_C_10_P_30_, and PC_90_C_10_P_50_ were determined and results are illustrated in Figure 2. Without the addition of PEO-7000K, there was instantaneous release of CPT11 with no delay from PC_90_C_10_P_0_. This can be explained by the fact that CPT11 is completely soluble in _LB_SNEP, which was able to self-nanoemulsify in the simulated gastric solution to completely solubilize the loaded drug leading to the instantaneous release of CPT11 into the dissolution medium. With mixing of increasing weight % of PEO-7000K in _LB_SNENP, a retardation of the initial released amount was seen with an increase in the weight % of PEO-7000K. In addition, it took about 2, 10, and 12 h for PC_90_C_10_P_10_, PC_90_C_10_P_30_, and PC_90_C_10_P_50_, respectively, to completely release CPT11 into the dissolution medium, confirming that sustained release of CPT11 was accomplished with the use of swellable PEO-7000K to form a hydrogel for sustained drug release. Due to limitations of the loading capacity of the 00 capsules, PC_90_C_10_P_10_ and PC_90_C_10_P_30_ were chosen for the following study.

### In vitro release of CPT11 and four dual-function inhibitors from optimal _LB_SNENPs

**Figure 3.**
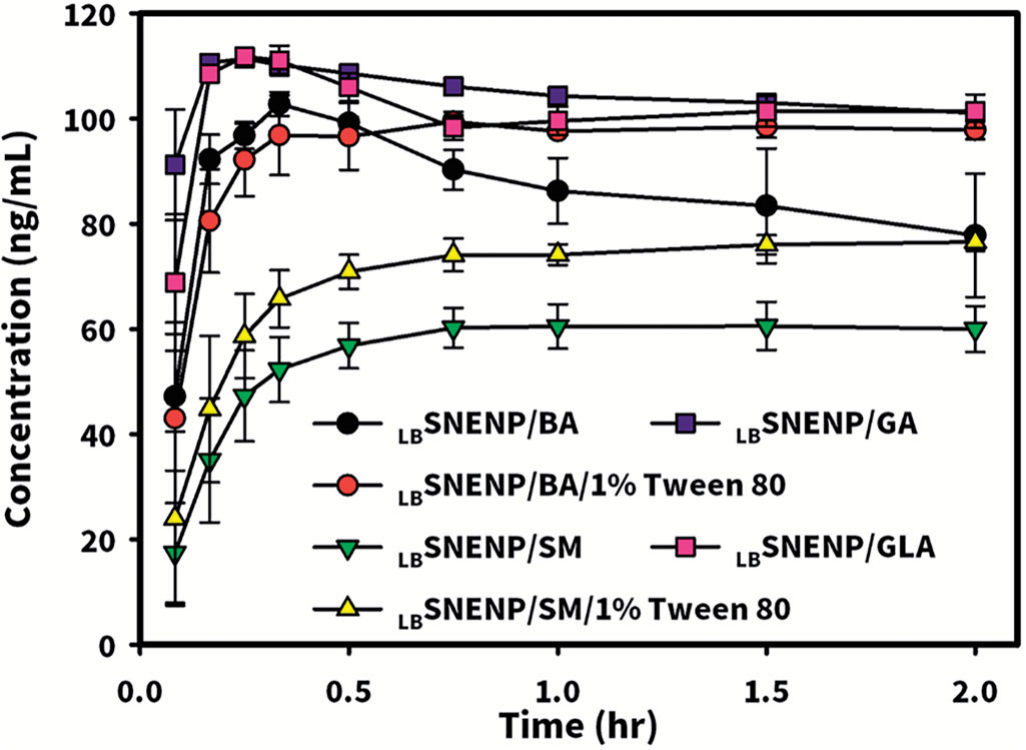
*In vitro* dissolution profiles of CPT11 (40 mg/g) and four dual-function inhibitors including BA, SM, GA, and GLA (80 mg/g) from PC_90_C_10_P_0_ performed using a simulated gastric acid solution with/without adding 1% Tween 80 as the dissolution medium. Each point represents the mean ± S.D. of three determinations (*n* = 3).

*In vitro* release of CPT11 (40 mg/g) and four dual-function inhibitors of BA, SM, GA, and GLA (80 mg/g) from PC_90_C_10_P_0_ were performed using a simulated gastric acid solution with/without adding 1% Tween 80 as the dissolution medium, and results are shown in Figure 3. The dissolution of CPT11, GA, and GLA from PC_90_C_10_P_0_ was observed to reach 100% within 0.5 h followed by maintenance at a plateau in the simulated gastric acid solution. However, the dissolution of BA from PC_90_C_10_P_0_ into the simulated gastric acid solution appeared to be over saturated, causing a gradual decrease in the BA concentration as a result of crystallization. Incomplete dissolution of SM from PC_90_C_10_P_0_ was shown which reached a plateau at 0.5 h with release of only 60%. When 1% Tween 80 was added to the simulated gastric acid solution as a solubilizer for BA and SM as the dissolution medium, a gradual decrease in the BA concentration as a result of crystallization from an oversaturated concentration was not observed for the dissolution of BA, and the amount of SM released at the plateau reached at 0.5 h increased to nearly 80%. Obviously, respective loading amounts of 40 and 80 mg/g of PC_90_C_10_P_0_ were workable for the oral delivery of CPT11 and four dual-function inhibitors in capsule dosage form. It was concluded that a combination of CPT11 at a loading amount of 40 mg/g of PC_90_C_10_P_0_ with four dual-function inhibitors at a loading amount of 80 mg/g of PC_90_C_10_P_0_ was feasible to examine their effects on the oral bioavailability and conversion efficiency of CPT11 to its active metabolite, SN-38.

### In vivo PK studies of CPT11 and SN-38 in rabbits

**Figure 4.**
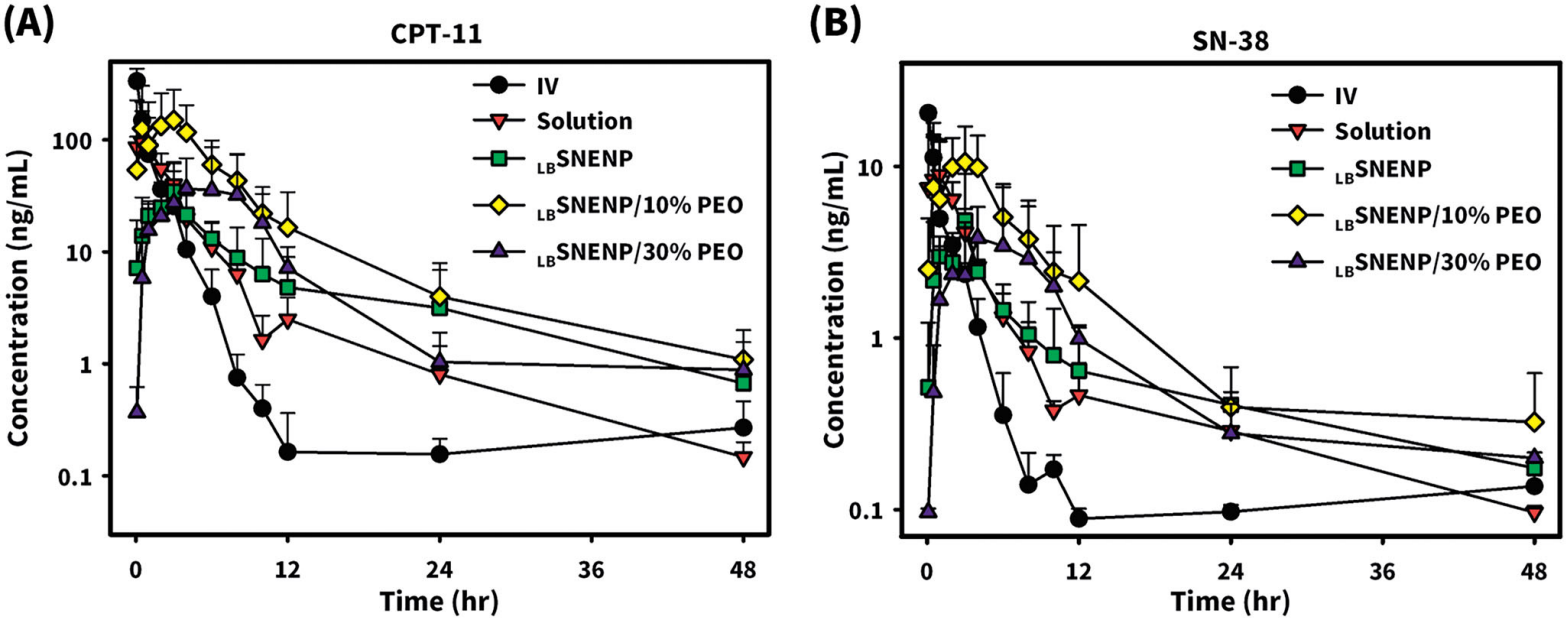
Plasma concentration profiles of CPT11 (A) and SN-38 (B) after oral administration of CPT11 (40 mg/per rabbit) solubilized in DD water (solution), PC_90_C_10_P_0_ (_LB_SNENP), PC_90_C_10_P_10_ (_LB_SNENP/10% PEO), and PC_90_C_10_P_30_ (_LB_SNENP/30% PEO) containing 10% and 30% PEO-7000K. Intravenous administration of CPT11 (IV) (20 mg/mL) at a dose of 4 mg/rabbit was included for calculation of the absolute bioavailability (*F*_AB_). Each point represents the mean ± S.D. of three determinations (*n* = 3).

**Figure 5.**
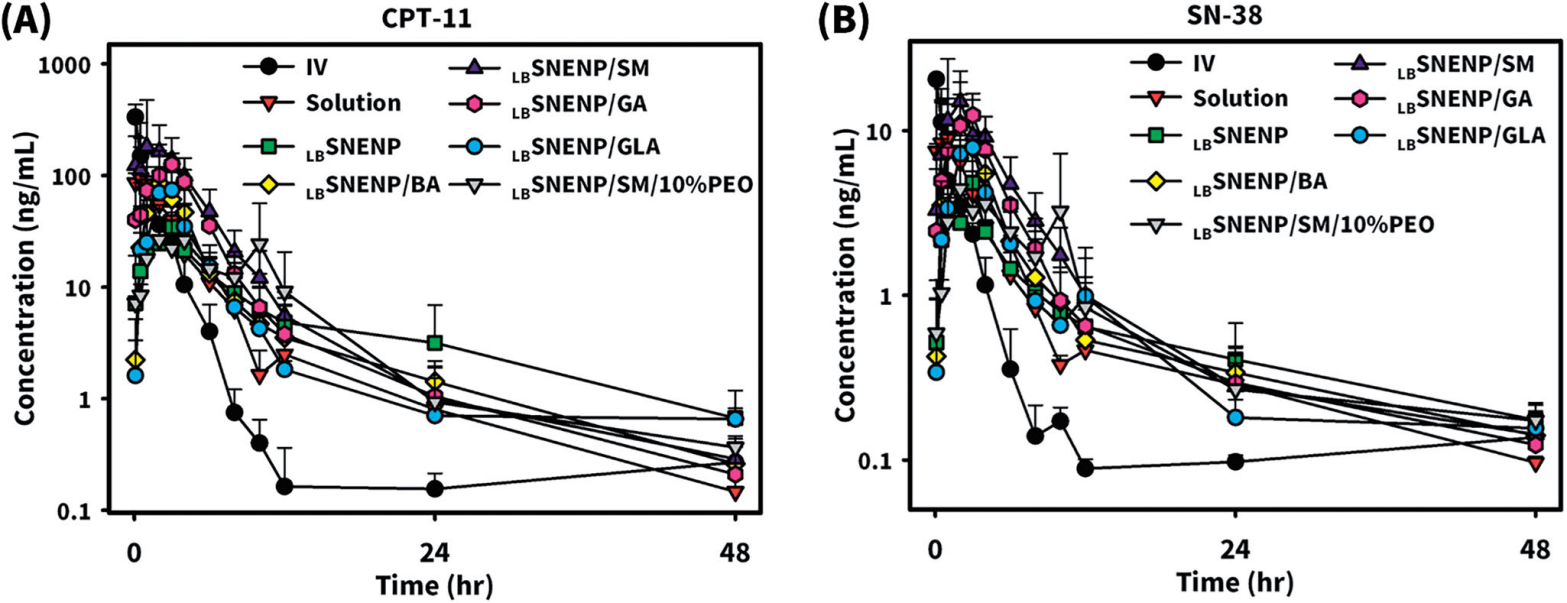
Plasma concentration profiles of CPT11 (A) and SN-38 (B) after oral administration of CPT11/four dual-function inhibitors co-loaded in PC_90_C_10_P_0_ (_LB_SNENP) (80 mg/rabbit) (_LB_SNENP/BA, _LB_SNENP/SM, _LB_SNENP/GA, and _LB_SNENP/GLA), or CPT11/SM co-loaded in PC_90_C_10_P_10_ (_LB_SNENP/SM/10% PEO). Intravenous administration of CPT11 (IV) (20 mg/mL) at a dose of 4 mg/rabbit was included for calculation of the absolute bioavailability. Each point represents the mean ± S.D. of three determinations (*n* = 3).

New Zealand white rabbits weighing ∼3 kg were utilized to investigate the PK profiles of CPT11 and SN-38 after the oral administration of CPT11 (40 mg/per rabbit) solubilized in DD water (solution), PC_90_C_10_P_0_ (_LB_SNENP), PC_90_C_10_P_10_ (_LB_SNENP/10% PEO), and PC_90_C_10_P_30_ (_LB_SNENP/30% PEO) containing 10% and 30% PEO-7000K. Plasma concentration profiles and related PK parameters for CPT11 and SN-38 were calculated, and results are illustrated in Figure 4(A,B) and listed in Tables 1 and 2, respectively. PK profiles of CPT11 and SN-38 were also sketched after oral administration of CPT11/four dual-function inhibitors co-loaded in PC_90_C_10_P_0_ (_LB_SNENP) (80 mg/ rabbit), in PC_90_C_10_P_0_ (_LB_SNENP/BA, _LB_SNENP/SM, _LB_SNENP/GA, _LB_SNENP/GLA), or CPT11/SM co-loaded in PC_90_C_10_P_10_ (_LB_SNENP/SM/10% PEO). Plasma concentration profiles and related PK parameters for CPT11 and SN-38 were calculated, and results are illustrated in Figure 5(A,B) and listed in Tables 3 and 4, respectively. For both PK studies, intravenous administration of CPT11 INJ (20 mg/mL) at a dose of 4 mg/rabbit was included for calculation of the absolute bioavailability.

**Table 1.** Pharmacokinetic parameters of CPT11 after administration of CPT11 in solution, _LB_SNEP (PC_90_C_10_P_0_), and _LB_SNEP with GRDDS (containing 10% or 30% PEO-7000K, PC_90_C_10_P_10_ and PC_90_C_10_P_30_) in rabbits.

Group	I.V. (4 mg/kg)	Solution	_LB_SNEP	_LB_SNEP 10% PEO	_LB_SNEP 30% PEO
*T*_max_ (h)	N/A	3.6 ± 0.9	2.2 ± 1.4	2.7 ± 0.6	6.3 ± 5.5
*C*_max_ (ng/mL)	341.0 ± 98.6	118.7 ± 110.8	36.5 ± 15.8	151.1 ± 128.5	43.5 ± 44.1
AUC_0–last_ (ng h/mL)	287.0 ± 103.9	318.1 ± 210.2	224.8 ± 27.3	994.1 ± 700.6	352.9 ± 288.6
AUC_0–∞_ (ng h/mL)	287.4 ± 104.1	322.6 ± 212.8	235.2 ± 27.2	1019.0 ± 683.9	365.7 ± 287.7
MRT (h)	2.4 ± 0.9	5.8 ± 1.4	11.8 ± 1.8	11.4 ± 6.8	14.7 ± 8.8
*T*_1/2_ (h)	8.1 ± 3.9	9.1 ± 3.6	12.7 ± 6.9	11.3 ± 2.7	11.5 ± 1.2
V (L)	163.0 ± 73.3	2045.7 ± 1174.5	3113.4 ± 1641.0	1133.9 ± 1218.7	2586.2 ± 1456.4
CL (L/h)	15.4 ± 5.61	160.5 ± 86.2	171.6 ± 20.3	61.9 ± 54.0	156.1 ± 92.4
*F*_AB_ (%)	100	11.0 ± 7.3	7.8 ± 1.0	34.6 ± 24.4	12.3 ± 10.1
*F*_RB1_ (%)	N/A	100	70.7 ± 8.6	312.5 ± 220.2*	110.9 ± 90.7*
*F*_RB2_ (%)	N/A	N/A	100	442.2 ± 311.7*	157.0 ± 128.4*

*Note*. Each point represents the mean ± S.D. of three determinations (*n* = 3).

*Significant (*p* < .05).

**Table 2.** Pharmacokinetic parameters of SN-38 after administration of CPT11 in solution, _LB_SNEP (PC_90_C_10_P_0_), and _LB_SNEP with GRDDS (containing 10% or 30% PEO-7000K, PC_90_C_10_P_10_ and PC_90_C_10_P_30_) in rabbits.

Group	I.V. (4 mg/kg)	Solution	_LB_SNEP	_LB_SNEP 10% PEO	_LB_SNEP 30% PEO
*T*_max_ (h)	0.1 ± 0.0	1.0 ± 1.0	1.8 ± 1.3	2.0 ± 1.0	5.7 ± 4.5
*C*_max_ (ng/mL)	19.85 ± 1.3	12.3 ± 7.6	5.6 ± 3.6	11.1 ± 5.7	4.3 ± 3.5
AUC_0–last_ (ng h/mL)	25.9 ± 8.2	42.4 ± 16.8	35.9 ± 7.8	95.4 ± 38.6	44.2 ± 19.3
AUC_0–∞_ (ng h/mL)	26.1 ± 8.3	45.0 ± 16.3	37.8 ± 8.1	105.3 ± 28.3	47.1 ± 18.5
MRT (h)	10.6 ± 1.7	11.3 ± 2.5	18.5 ± 2.3	16.5 ± 8.5	19.1 ± 7.1
*T*_1/2_ (h)	2.8 ± 0.6	13.4 ± 1.2	7.3 ± 3.8	13.8 ± 5.0	12.8 ± 5.0
*F*_AB_ (%)	100	16.4 ± 6.5	13.9 ± 3.0	36.8 ± 14.9	17.1 ± 7.5
*F*_RB1_ (%)	N/A	100	84.7 ± 18.4	225.0 ± 91.0*	104.2 ± 45.5
*F*_RB2_ (%)	N/A	N/A	100	265.7 ± 107.5*	123.1 ± 53.8
Conversion efficiency (%)	9.0 ± 2.9	13.3 ± 5.3	16.0 ± 3.5	9.5 ± 3.9	12.5 ± 5.5

*Note*. Each point represents the mean ± S.D. of three determinations (*n* = 3).

*Significant (*p* < .05).

**Table 3.** Pharmacokinetic parameters of CPT11 after administration of CPT11 and four dual-function inhibitors co-loaded _LB_SNEP (PC_90_C_10_P_0_) and CPT11/silymarin co-loaded _LB_SNEP/10% PEO-7000K (PC_90_C_10_P_10_) in rabbits.

Group	_LB_SNEP Baicalein	_LB_SNEP Silymarin	_LB_SNEP GA	_LB_SNEP GLA	_LB_SNEP/10%PEO Silymarin
*T*_max_ (h)	2.7 ± 1.2	1.7 ± 0.6	2.0 ± 1.0	2.7 ± 0.6	7.0 ± 4.2
*C*_max_ (ng/mL)	98.1 ± 93.8	292.1 ± 228.5	140.7 ± 16.9	81.2 ± 43.5	46.7 ± 0.5
AUC_0–last_ (ng × h/mL)	345.4 ± 250.5	832.0 ± 401.2	579.1 ± 77.8	305.4 ± 165.3	272.6 ± 36.6
AUC_0–∞_ (ng × h/mL)	350.3 ± 253.2	840.1 ± 389.5	580.7 ± 79.4	307.5 ± 166.4	274.5 ± 36.0
MRT (h)	8.0 ± 2.8	6.6 ± 4.1	5.0 ± 1.0	7.3 ± 0.9	9.0 ± 2.9
*T*_1/2_ (h)	9.9 ± 3.0	9.9 ± 1.1	10.6 ± 1.4	10.4 ± 4.7	4.2 ± 1.0
V (L)	2229.3 ± 1691.7	802.7 ± 413.3	1054.6 ± 79.7	2120.2 ± 893.3	851.5 ± 362.9
CL (L/h)	155.4 ± 89.5	58.6 ± 35.8	69.8 ± 10.0	155.5 ± 72.8	135.8 ± 27.5
*F*_AB_ (%)	12.0 ± 8.7	28.9 ± 14.0	20.2 ± 2.7	10.6 ± 5.8	9.5 ± 1.3
*F*_RB1_ (%)	108.6 ± 78.7	261.6 ± 126.1*	182.1 ± 24.5*	96.0 ± 52.0	85.7 ± 11.5
*F*_RB2_ (%)	153.6 ± 111.3	370.1 ± 178.5*	257.6 ± 34.6*	135.9 ± 73.5	121.3 ± 16.3

*Note*. Each point represents the mean ± S.D. of three determinations (*n* = 3).

*Significant (*p* < .05).

**Table 4.** Pharmacokinetic parameters of SN-38 after oral administration of CPT11 and four dual-function inhibitors co-loaded _LB_SNEP (PC_90_C_10_P_0_) and CPT11/silymarin co-loaded _LB_SNEP containing 10% PEO-7000K (PC_90_C_10_P_10_) in rabbits.

Group	_LB_SNEP Baicalein	_LB_SNEP Silymarin	_LB_SNEP GA	_LB_SNEP GLA	_LB_SNEP/10%PEO Silymarin
*T*_max_ (h)	2.7 ± 1.2	1.7 ± 0.6	2.3 ± 0.6	27 ± 0.6	6.0 ± 5.7
*C*_max_ (ng/mL)	12.3 ± 10.6	18.2 ± 10.1	13.±3.3	7.9 ± 1.5	6.8 ± 1.0
AUC_0–last_ (ng h/mL)	55.2 ± 31.5	84.3 ± 15.9	66.9 ± 6.4	45.0 ± 11.0	41.3 ± 1.4
AUC_0–∞_ (ng h/mL)	58.5 ± 33.1	87.3 ± 15.7	68.9 ± 7.5	47.4 ± 10.5	42.9 ± 3.0
MRT (h)	12.5 ± 3.4	6.6 ± 2.7	8.9 ± 2.1	12.4 ± 1.6	10.3 ± 3.7
*T*_1/2_ (h)	10.8 ± 5.4	12.5 ± 5.3	11.1 ± 5.4	13.7 ± 4.7	5.7 ± 4.8
*F*_AB_ (%)	21.3 ± 12.2	32.5 ± 6.1	25.8 ± 2.5	17.4 ± 4.2	15.9 ± 0.5
*F*_RB1_ (%)	130.2 ± 74.3*	198.8 ± 37.5*	157.8 ± 15.1*	106.1 ± 25.9	97.4 ± 3.3
*F*_RB2_ (%)	153.8 ± 59.9*	234.8 ± 44.3*	186.4 ± 17.8*	125.3 ± 30.6	115.0 ± 3.9
Conversion efficiency (%)	16.0 ± 9.1	10.1 ± 1.9	11.6 ± 1.1	14.7 ± 3.6	15.2 ± 0.5

*Note*. Each point represents the mean ± S.D. of three determinations (*n* = 3).

*Significant (*p* < .05).

Plasma concentration profiles of CPT11 as shown in Figure 4(A) and calculated PK parameters as listed in Table 1 demonstrated that the oral administration of CPT11 solubilized in solution resulted in a *T*_max_ of 3.6 ± 0.9 h, *C*_max_ of 118.7 ± 110.8 ng/mL, AUC_0-last_ of 318.1 ± 210.2 ng·h/mL, *T*_1/2_ of 9.1 ± 3.6 h, and MRT of 5.8 ± 1.4 h, with an absolute bioavailability (*F*_AB_) of 11.0 ± 7.3% (refers to i.v. administration of CPT11) with a greater extent of variation, whereas respective values with the oral administration of CPT11 loaded in _LB_SNENP (PC_90_C_10_P_0_) were observed to be 2.2 ± 1.4 h, 36.5 ± 15.8 ng/mL, 224.8 ± 27.3 ng·h/mL, 12.7 ± 6.9 h, and 11.8 ± 1.8 h with an absolute bioavailability (*F*_AB_) of 7.8 ± 1.01% (refers to i.v. administration of CPT11) with a lower extent of variation and a relative bioavailability (*F*_RB1_) of 70.7 ± 8.6% (refers to oral administration of the CPT11 solution). Although loading CPT11 in _LB_SNENP (PC_90_C_10_P_0_) did not enhance the oral bioavailability, a longer *T*_1/2_ (12.7 ± 6.9 vs. 9.1 ± 3.6 h) and MRT (11.8 ± 1.8 vs. 5.8 ± 1.4 h) implied that longer exposure of the tumor to CPT11 circulating in the blood would result from the oral administration of CPT11 loaded in _LB_SNENP (PC_90_C_10_P_0_), potentially leading to increased therapeutic efficacy.

Plasma concentration profiles of CPT11 as shown in Figure 4(A) and calculated PK parameters listed in Table 1 further demonstrated that oral administration of CPT11 loaded in _LB_SNENP with the addition of 10% PEO-7000K (PC_90_C_10_P_10_) resulted in a *T*_max_ of 2.7 ± 0.6 h, *C*_max_ of 151.1 ± 128.5 ng/mL, AUC_0-last_ of 994.1 ± 700.6 ng·h/mL, *T*_1/2_ of 11.4 ± 6.8 h, and MRT of 11.3 ± 2.7 h, with *F*_AB_ of 34.6 ± 24.4% and *F*_RB1_ of 312.5 ± 220.2%, while respective values for CPT11 loaded in _LB_SNENPs with the addition of 30% PEO-7000K (PC_90_C_10_P_30_) were 6.3 ± 5.5 h, 43.5 ± 44.1 ng/mL, 352.9 ± 288.6 ng·h/mL, 14.7 ± 8.8 h, and 11.5 ± 1.2 h, with *F*_AB_ of 12.3 ± 10.1% and *F*_RB1_ of 110.9 ± 90.7%. Results revealed that incorporation of PEO-7000K into the GRDDS caused CPT11-loaded _LB_SNENPs (PC_90_C_10_P_10_ and PC_90_C_10_P_30_) to be retained in the stomach with an appropriate sustained release rate of CPT11 leading to the enhancements of *C*_max_, AUC_0-last_, and *F*_RB1_ (312.5 ± 220.2% and 110.9 ± 90.7%) similarly with a longer *T*_1/2_ (11.4 ± 6.8 vs. 9.1 ± 3.6 h; 14.7 ± 8.8 vs. 9.1 ± 3.6 h) and MRT (11.8 ± 1.8 vs. 5.8 ± 1.4; 11.5 ± 1.2 vs. 5.8 ± 1.4 h) for both PC_90_C_10_P_10_ and PC_90_C_10_P_30_. Furthermore, values of *F*_RB2_ for PC_90_C_10_P_10_ and PC_90_C_10_P_30_ were 4.0-fold (442.2 ± 311.7%) and 1.5-fold higher (157.0 ± 128.4%), respectively, than those for PC_90_C_10_P_0_, which did not incorporate PEO-7000K. This indicates that incorporation of CPT11-loaded _LB_SNENP in GRDDSs can promote the oral bioavailability as a result of CPT11 being released in the acidic pH environment to ensure that CPT11 is maintained in its active lactone form and prevent it from transitioning to the lower part of GI tract, whereby efflux by P-gp decreasing the bioavailability could be avoided. Nevertheless, the increase in PEO-7000K from 10% to 30% did not proportionally enhance the oral bioavailability compared to PC_90_C_10_P_0_. This might be explained by the increase in the added amount of the hydrophilic PEO-7000K polymer could absorb most of water taken with the medicine leaving no water available for dissolution, and also resulted in the hydrogel formed being too viscous to retard the release rate of CPT11 from the obtained hydrogel matrix, both of which caused a decrease in the oral bioavailability. As expected, both the enhancement of oral bioavailability and establishment of a longer *T*_1/2_ and MRT profoundly indicated that after oral administration of CPT11-loaded PC_90_C_10_P_10_ and PC_90_C_10_P_30_, tumors would be exposed to higher CPT11 concentrations circulating in the blood for longer periods of time potentially leading to enhancement of the therapeutic efficacy compared to that for CPT11 solubilized in water and CPT11 loaded in _LB_SNEPs (PC_90_C_10_P_0_).

Since CPT11 is metabolized into its major active (100–1000 times) metabolite of SN-38 with the help of carboxylesterases that are located in enterocytes and hepatocytes but is subjected to a competing process with the CYP3A oxidation of CPT11 into the inactive metabolites of APC and NPC, the extent of formation of SN-38 after oral administration of CPT11 plays a determining role in its therapeutic efficacy. Plasma concentration profiles of the formation of SN-38 in rabbits after oral administration of CPT11 solubilized in DD water (solution), PC_90_C_10_P_0_ (_LB_SNENP), PC_90_C_10_P_10_ (_LB_SNENP/10% PEO), and PC_90_C_10_P_30_ (_LB_SNENP/30% PEO) containing 10% and 30% PEO-7000K, respectively, are shown in Figure 4(B), and calculated PK parameters are listed in Table 2. Results demonstrated that oral administration of CPT11 solubilized in solution resulted in the formation of SN-38 with a *T*_max_ of 1.0 ± 1.0 h, *C*_max_ of 12.3 ± 7.6 ng/mL, AUC_0-last_ of 42.4 ± 16.8 ng·h/mL, *T*_1/2_ 13.4 ± 1.2 h, and MRT of 11.3 ± 2.5 h, with *F*_AB_ of 16.4 ± 6.5% and a conversion efficiency of 13.3 ± 5.3%, while respective values for the oral administration of CPT11 loaded in _LB_SNENP (PC_90_C_10_P_0_) observed were 1.8 ± 1.3 h, 5.6 ± 3.6 ng/mL, 35.9 ± 7.8 ng·h/mL, 7.3 ± 3.8 h, and 18.5 ± 2.3 h with *F*_AB_ of 13.9 ± 3.0%, *F*_RB1_ of 84.7 ± 18.4%, and a conversion efficiency of 16.0 ± 3.5%. Although the extent of formation of SN-38 after oral administration of CPT11 loaded in _LB_SNENP (PC_90_C_10_P_0_) showed no enhancement relative to that for oral administration of CPT11 in solution, its higher conversion efficiency of 16.0 ± 3.5% with a longer MRT (18.5 ± 2.3 vs. 11.3 ± 2.5 h) means that longer exposure to SN-38 that was converted from the absorbed CPT11 after oral administration would be expected, potentially leading to improved therapeutic efficacy.

Results shown in Figure 4(B) and Table 2 further demonstrated that oral administration of CPT11 solubilized in PC_90_C_10_P_10_ (_LB_SNENP/10% PEO) resulted in the formation of SN-38 with a *T*_max_ of 2.0 ± 1.0 h, *C*_max_ of 11.1 ± 5.7 ng/mL, AUC_0-last_ of 95.4 ± 38.6 ng·h/mL, *T*_1/2_ of 13.8 ± 5.0 h, and MRT of 16.5 ± 8.5 h, with *F*_AB_ of 36.8 ± 14.9%, *F*_RB1_ of 225.0 ± 91.0%, and a conversion efficiency of 9.5 ± 3.9%, while values for oral administration of CPT11 loaded in PC_90_C_10_P_30_ (_LB_SNENP/30% PEO) were 5.7 ± 4.5 h, 4.3 ± 3.5 ng/mL, 44.2 ± 19.3 ng·h/mL, 12.8 ± 5.0 h, and 19.1 ± 7.1 h with *F*_AB_ of 17.1 ± 7.5%, *F*_RB1_ of 104.2 ± 45.5%, and a conversion efficiency of 12.5 ± 5.5%. Even though there was a lower conversion efficiency of the formation of SN-38 for the oral administration of CPT11 solubilized in both PC_90_C_10_P_10_ (_LB_SNENP/10% PEO) and PC_90_C_10_P_30_ (_LB_SNENP/30% PEO) compared to that for the oral administration of CPT11 loaded in PC_90_C_10_P_0_ (_LB_SNENP), a longer exposure (longer MRT) to a higher concentration of SN-38 (larger AUC) for both would be expected, and similarly there would be a greater therapeutic efficacy, exceptionally with the oral administration of CPT11 solubilized in PC_90_C_10_P_10_ (_LB_SNENP/10% PEO).

Plasma concentration profiles of CPT11 are shown in Figure 5(A) and calculated PK parameters are listed in Table 3 revealing results of the oral administration of CPT11-loaded _LB_SNENPs (PC_90_C_10_P_0_) combined with four dual-function inhibitors (BA, SM, GA, and GLA) and CPT11/SM-loaded _LB_SNEPs with the addition of 10% PEO-7000K (PC_90_C_10_P_10_). Results demonstrated that the order of *F*_RB1_ values for CPT11-loaded _LB_SNENPs (PC_90_C_10_P_0_) combined with BA, SM, GA, and GLA was as follows, SM (261.6 ± 126.1%) > GA (182.1 ± 24.5%) > BA (108.6 ± 78.7%) > GLA (96.0 ± 52.0%) with only that for GLA being lower than 100%. Furthermore, the order of *F*_RB2_ values of CPT11 in _LB_SNENP (PC_90_C_10_P_0_) combined with BA, SM, GA, and GLA was as follows, SM (370.1 ± 178.5%) > GA (257.6 ± 34.6%) > BA (153.6 ± 111.3%) > GLA (135.9 ± 73.5%) with all being greater than 100%. This indicates that SM as a dual-functional inhibitor showed the most profound influence on the oral bioavailability of CPT11 when it was loaded with CPT11 in _LB_SNENP (PC_90_C_10_P_0_). However, CPT11/SM-loaded _LB_SNEPs with the addition of 10% PEO-7000K (PC_90_C_10_P_10_) only resulted in a *F*_RB1_ of 85.7 ± 11.5% and a *F*_RB2_ of 121.3 ± 16.3%, which was just 20% higher than that for the oral administration of CPT11 loaded in the plain _LB_SNENP (PC_90_C_10_P_0_). It was suspected that the release of SM from the viscous hydrogel formed with the capsule containing CPT11-loaded PC_90_C_10_P_10_ fell behind that of CPT11 which did not cause inhibition of P-gp and CYP-3A4 present in absorption sites along the GIT and before CPT11 reached the first-pass effect. It might be concluded that the combination of SM as a dual-function inhibitor with CPT11 in plain _LB_SNENPs (PC_90_C_10_P_0_) resulted in significant 2.6- and 3.7-fold increases, respectively, in the relative bioavailability (*F*_RB1_) compared to that of the CPT11 solution and the relative bioavailability (*F*_RB2_) relative to that of CPT11-loaded plain _LB_SNENPs (PC_90_C_10_P_0_). However, the combination of SM as a dual-function inhibitor with CPT11 in PEO-7000K-containing _LB_SNENPs (PC_90_C_10_P_0_) only resulted in a fair enhancement of both *F*_RB1_ and *F*_RB2,_ and showed less improvement in the oral bioavailability of CTP-11 in the presence of dual-function inhibitors.

Plasma concentration profiles of the formation of SN-38 in rabbits after oral administration of CPT11-loaded _LB_SNENPs (PC_90_C_10_P_0_) combined with four dual-function inhibitors (BA, SM, GA, and GLA) in _LB_SNENPs (PC_90_C_10_P_0_) and CPT11/SM-loaded _LB_SNEPs with the addition of 10% PEO-7000K (PC_90_C_10_P_10_) are shown in Figure 5(B), and calculated PK parameters are listed in Table 4. Results demonstrated that the order of oral bioavailability (*F*_RB1_) for the formation of SN-38 for CPT11 combined with BA, SM, GA, and GLA in PC_90_C_10_P_0_ was as follows, SM (198.8 ± 37.5%) > GA (157.8 ± 15.1%) > BA (130.2 ± 74.3%) > GLA (106.1 ± 25.9%). Conversion efficiencies for combining BA, SM, GA, and GLA with the oral administration of CPT11-loaded _LB_SNENP (PC_90_C_10_P_0_) were 16.0 ± 9.1%, 10.1 ± 1.9%,11.6 ± 1.1%, and 14.7 ± 3.6%, respectively. This indicated that SM as a dual-function inhibitor showed the most profound enhancement of the oral bioavailability of CPT11 when it was loaded in _LB_SNENPs (PC_90_C_10_P_0_), in turn increasing the extent of formation of SN-38 with the lowest conversion efficiency among the four dual-function inhibitors. However, combining SM with the oral administration of CPT11-loaded _LB_SNEPs with the addition of 10% PEO-7000K (PC_90_C_10_P_10_) only resulted in a *F*_RB1_ of 97.4 ± 3.3% with a conversion efficiency of 15.2 ± 0.5%, which was lower in terms of *F*_RB1_ but higher in conversion efficiency than those with the oral administration of CPT11 loaded in the plain _LB_SNENPs (PC_90_C_10_P_0_). It was concluded that combining SM as a dual-function inhibitor with the oral administration of CPT11-loaded _LB_SNENPs (PC_90_C_10_P_0_) could efficaciously enhance the oral bioavailability of CPT11 with a 2-fold increase in the formation of the active metabolite, SN-38, even though with only a moderate conversion efficiency. This also implied that CPT11 combined with SM solubilized in _LB_SNENPs (PC_90_C_10_P_0_) might improve the therapeutic efficacy against tumors to the highest extent compared to CPT11 loaded in _LB_SNENPs (PC_90_C_10_P_0_) combined with or without the three other dual-function inhibitors as a result of exposure to higher concentrations of both CPT11 and its active metabolite, SN-38.

### In vivo tumor growth inhibition (TGI) studies in mice

**Figure 6.**
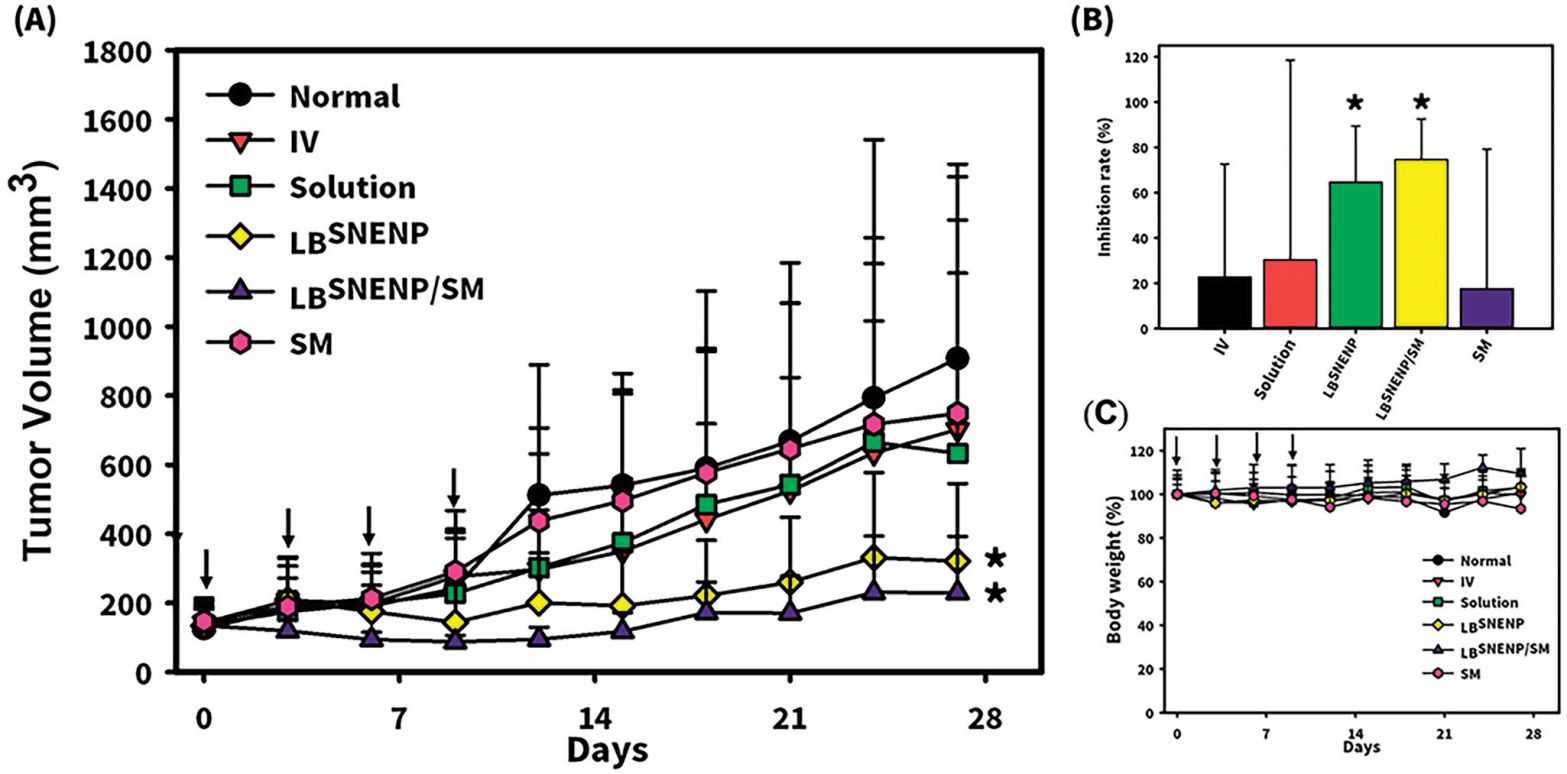
Antitumor effects of the oral administration of CPT11 alone in water by injection, SM alone solubilized in _LB_SNENP (PC_90_C_10_P_0_), CPT11 solubilized in _LB_SNENP (PC_90_C_10_P_0_), and CPT11 combined with SM in _LB_SNENP (PC_90_C_10_P_0_) with two control groups of the oral administration of a PBS solution and i.v. administration of a CPT11 solution were evaluated in an MIA PaCa-2 xenograft mouse model. (A) Tumor growth curves; (B) tumor weights measured at the end of the study; (C) profiles of body weight changes of mice after administration. Each point represents the mean ± S.D. of three determinations (*n* = 5). *Significant (*p* < .05).

Since CPT11 combined with SM was found to produce profound improvements in the oral bioavailability of CPT11 and the formation of its active metabolite, SN-38, and its incorporation with PEO-7000K were not beneficial in improving the oral bioavailability of CPT11 or the formation of its active metabolite, SN-38, only SM alone solubilized in _LB_SNENPs (PC_90_C_10_P_0_), CPT11 solubilized in _LB_SNENPs (PC_90_C_10_P_0_), and CPT11 combined with SM in _LB_SNENPs (PC_90_C_10_P_0_) were included in the TGI studies. The antitumor effects of the oral administration of CPT11 alone in water by injection, SM alone solubilized in _LB_SNENPs (PC_90_C_10_P_0_), CPT11 solubilized in _LB_SNENPs (PC_90_C_10_P_0_), and CPT11 combined with SM in _LB_SNENPs (PC_90_C_10_P_0_) with two control groups of the oral administration of a PBS solution and the i.v. administration of a CPT11 solution were evaluated in an MIA PaCa-2 xenograft mouse model, and results of tumor growth profiles and weight change profiles are respectively presented in Figure 6(A,B). Results shown in Figure 6(A) clearly demonstrate that only CPT11 solubilized in _LB_SNENPs (PC_90_C_10_P_0_) and CPT11 combined with SM in _LB_SNENPs (PC_90_C_10_P_0_) efficaciously inhibited the growth of MIA PaCa-2 tumors after treatment with a regimen of 50 mg/kg for Q3*4. Furthermore, as shown in Figure 6(B), the TGI rate (%) after treatment with i.v. administration of the CPT11 solution, and oral administration of CPT11 alone in water by injection, SM alone solubilized in _LB_SNENPs (PC_90_C_10_P_0_), CPT11 solubilized in _LB_SNENPs (PC_90_C_10_P_0_), and CPT11 combined with SM in _LB_SNENPs (PC_90_C_10_P_0_) calculated with respect to that for the PBS treatment group (as a baseline) were 22.70 ± 49.95%, 17.55 ± 61.67%, 30.28 ± 88.20%, 64.65 ± 24.75%, and 74.67 ± 17.89%, respectively. Formulations of CPT11 solubilized in _LB_SNENPs (PC_90_C_10_P_0_) and CPT11 combined with SM in _LB_SNENPs (PC_90_C_10_P_0_) both showed the greatest antitumor effects with the latter slightly better than the former, and tumors were significantly suppressed compared to the control group of PBS (*p* < .05). Furthermore, the weight change profiles of all treatment groups illustrated in Figure 6(C) demonstrate that there was no more than 20% weight loss observed in any treatment groups, indicating that all formulations induced little systemic toxicity.

As discussed above, although the oral administration of CPT11 loaded in _LB_SNENPs (PC_90_C_10_P_0_) did not enhance the oral bioavailability compared to that for CPT11 solubilized in solution and the extent of formation of SN-38 after oral administration of CPT11 loaded in _LB_SNENPs (PC_90_C_10_P_0_) also showed no enhancement relative to that for the oral administration of CPT11 solubilized in solution, a longer *T*_1/2_ (12.7 ± 6.9 vs. 9.1 ± 3.6 h) and MRT (11.8 ± 1.8 vs. 5.8 ± 1.4 h) for those with absorbed CPT11 and its higher conversion efficiency of 16.0 ± 3.5% to SN-38 with a longer MRT (18.5 ± 2.3 vs. 11.3 ± 2.5 h) after oral administration of CPT11 loaded in _LB_SNENPs (PC_90_C_10_P_0_) mean that a longer exposure to both CPT11 and SN-38 would be expected, potentially leading to improved therapeutic efficacy as results of the TGI study demonstrated. Regarding combining SM as a dual-function inhibitor in _LB_SNENPs (PC_90_C_10_P_0_), the oral bioavailability of CPT11 relative to that of only CPT11 loaded in _LB_SNENPs (PC_90_C_10_P_0_) showed the most profound enhancement of 261.6 ± 126.1% with a 2-fold increase in the formation of the active metabolite, SN-38, even with a moderate conversion efficiency. This could be explained as the therapeutic efficacy against tumors after oral administration of CPT11 combined with SM loaded in _LB_SNENPs (PC_90_C_10_P_0_) improved to a greater or lesser extent compared to those for both only CPT11 loaded in solution and in _LB_SNENPs (PC_90_C_10_P_0_).

## Conclusions

It was concluded that oral treatment with CPT11 loaded in lecithin-based self-nanoemulsifying nanoemulsion preconcentrates (_LB_SNENPs) composed of Capryol 90, SAA, and PG at a weight ratio of 18:58:24 (PC_90_C_10_P_0_) with or without solubilization of SM could expose tumors to higher plasma concentrations of both CPT11 and its active metabolite, SN-38, leading to enhanced inhibition of tumor growth with no signs of adverse effects. It also proved that an optimal lower dose of CPT11 with a desirable oral administration regimen could potentially provide a greater efficacy of extended-duration therapy with reduced toxicity. Metronomic chemotherapy of tumors with the oral administration of CPT11 at lower doses with a more-frequent regimen to reduce toxicity and resolve tolerability is worthy of clinical trials.
